# Acid sphingomyelinase deficiency with homozygous p.Arg610del genotype in an elderly patient: a rare case report

**DOI:** 10.36416/1806-3756/e20240192

**Published:** 2024-11-16

**Authors:** Guilherme das Posses Bridi, Ronaldo Adib Kairalla, Márcio Valente Yamada Sawamura, Bruno Guedes Baldi

**Affiliations:** 1. Divisao de Pneumologia, Instituto do Coracao, Hospital das Clinicas - HCFMUSP - Faculdade de Medicina, Universidade de Sao Paulo, Sao Paulo (SP) Brasil.; 2. Instituto de Radiologia, Hospital das Clínicas - HCFMUSP - Faculdade de Medicina, Universidade de São Paulo, São Paulo (SP) Brasil.

## TO THE EDITOR:

Acid sphingomyelinase deficiency (ASMD; alternatively known as Niemann-Pick disease types A, B, and A/B) is an ultra-rare lysosomal storage multisystemic disorder caused by pathogenic variants of the sphingomyelin phosphodiesterase 1 (*SMPD1*) gene with over 250 variants described in ASMD patients. The global prevalence is estimated at approximately 1:100,000-1,000,000 births.^1^ Niemann-Pick types A and B are pan-ethnic, but it is well-documented that type A-causing *SMPD1* variants occur more frequently among individuals of Ashkenazi Jewish ancestry than in the general population. The true incidence of type B disease in many countries remains unknown, frequently underdiagnosed by clinicians.^2^ Clinical manifestations and disease severity can vary according to the subtype of disease, and predominant genetic alteration. The pathophysiology of the disease is yet to be completely understood, but initial accumulation of lysosomal sphingomyelin leads to inflammatory changes and apoptosis, mitochondrial defects with impaired cellular respiration, and changes in nuclear transport.^1^ The disease spectrum ranges from infants with ASMD type A, who have a short life expectancy and rapidly progressive neurodegeneration, to more chronic and variable ASMD types A/B and B, which have a more slowly progressive visceral and/or neurovisceral disease.^3^

The most common symptoms in chronic progressive disease are splenomegaly, hepatomegaly, interstitial lung disease with frequent respiratory infections, high total cholesterol and triglyceride levels-which may lead to cardiac disease-liver fibrosis, cytopenias, including thrombocytopenia with bleeding episodes, and osteopenia or osteoporosis. However, the occurrence of such clinical manifestations is heterogeneous. Historically, the diagnosis of ASMD is challenging, and the disease is certainly underestimated. When there is a suspicion of ASMD, it is important to conduct an enzyme assay to assess ASM activity. The diagnosis is confirmed by showing that ASM activity is deficient or markedly reduced in leukocytes, fibroblasts, or dried blood spots. Assessment of mutations in the *SMPD1* gene should be performed to confirm the diagnosis of ASMD in subjects with ASM activity below the normal reference values.^1^ ^,^ ^3^

**Figure 1:**
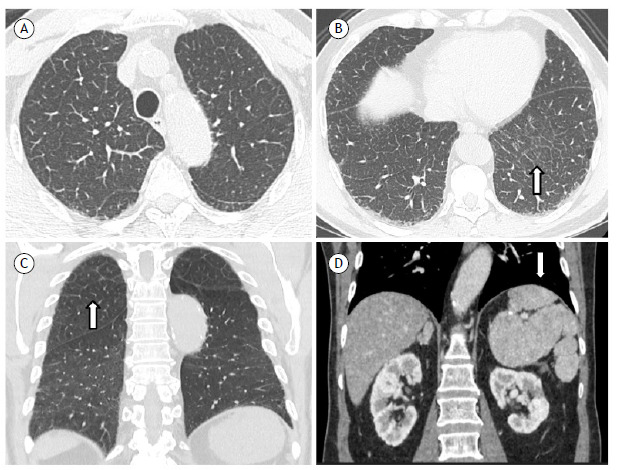
Chest CT shows diffuse septal thickening (arrows) and mild areas of emphysema predominantly in the upper lung lobes in A and B (axial reconstruction) and C (coronal reconstruction). In D, coronal reconstruction shows the remaining spleen (arrow).

We present here a case of a 77-year-old man, White, who was admitted to our outpatient clinic with dyspnea and hypoxemia after adrenalectomy. He had adrenal macronodular hyperplasia with Cushing’s syndrome and ischemic heart disease with hypertriglyceridemia. He was a former smoker. Additionally, he underwent splenectomy due to splenomegaly 30 years prior. At clinical evaluation, he had dyspnea on exertion, oxygen saturation of 92% on room air, and fine crackles in the lower lobes. Chest CT showed diffuse septal thickening with mild areas of emphysema predominantly in the upper lung lobes (Figures 1A, 1B, and 1C) and a remaining spleen was identified (Figure 1D). Pulmonary function tests demonstrated a proportional reduction in FEV_1_ and FVC (FVC = 76% of predicted), normal TLC values (83% of predicted), and a severe reduction in DL_CO_ (41% of predicted). Laboratory tests were unremarkable, including negative results for autoantibodies such as antinuclear antibody, rheumatoid factor, anti-Ro/SSA, and La/SSB. Liver enzymes were normal, and there was no thrombocytopenia. The ASM activity level measured was 0.37 µmol/L/h (normal value > 1.02), and genetic testing confirmed the homozygous *p.Arg610del* mutation, establishing the diagnosis of ASMD.

This is a rare case report of an elderly patient with ASMD with the *p.Arg610del* variant in homozygosity and adrenal macronodular hyperplasia. Gene sequencing is recommended for ASMD patients, because genotyping may predict the disease course, chronic visceral manifestations, and particularly, the likelihood of neurodegeneration and/or type A disease.^1^ ^,^ ^4^ The *p.Arg610del* pathogenic variant is associated with the absence of neurodegenerative disease in both homozygous and compound heterozygous patients. Patients who are homozygous for this “neuroprotective” variant have a milder disease course, normal growth, and normal bone development in comparison with those who are *p.Arg610del* heterozygous and those with other genotypes.^4^ This mutation has been described to be associated with an attenuated phenotype of ASMD type B disease, being one of the most common associated variants.^5^ ^,^ ^6^ In our case, the diagnosis was late, there were no neurological manifestations or severe pulmonary involvement, and the patient remained stable over the years, with no clinical complications.

ASMD is an autosomal recessive disease that results from the reduced activity of ASM due to loss-of-function variants in *SMPD1*. Over 250 *SMPD1* variants have been described worldwide, resulting in different clinical phenotypes. ASMD is a chronic condition in which the main therapy is management of complications and symptoms.^1^ Olipudase alfa, an enzyme replacement therapy, is a promising drug for ASMD, and recent studies and series have demonstrated improvement in sphingomyelin storage, organomegaly, interstitial lung disease, and DL_CO_, showing a safe profile after two years.^7^ ^,^ ^8^ Patients with ASMD eligible to receive olipudase alfa should be evaluated individually for the potential benefits on respiratory symptoms and pulmonary function tests, spleen and liver volume, platelet count, serum lipid profile, growth in children, and quality of life.^1^ In this case, the patient remained clinically and functionally stable during follow-up, with no other systemic manifestations, therefore, with no indication for olipudase alfa replacement.
